# Early post-operative CRP is a better predictor of DAIR failure than pre-operative CRP in total knee PJI

**DOI:** 10.1007/s00402-026-06369-2

**Published:** 2026-06-26

**Authors:** Harrison Beadel, Ryan Chaffey, Katy Kim, Mark Zhu, Brendan Coleman

**Affiliations:** Health New Zealand, Auckland, New Zealand

**Keywords:** Orthopaedics, PJI, DAIR, CRP, Arthroplasty, Infection

## Abstract

**Introduction:**

Debridement, antibiotics and implant retention (DAIR) surgery is often performed as a first-line treatment for periprosthetic joint infection (PJI), however, success rates are variable. This study aimed to determine whether post-operative C-reactive protein (CRP) levels predict DAIR failure.

**Materials and methods:**

A multi-centre retrospective cohort study was performed over a 15-year period. All total knee arthroplasty (TKA) patients undergoing DAIR for first episode PJI were included. CRP was measured at admission and for 6 weeks post-operatively. Treatment success was defined as implant retention without the need for revision surgery or long-term suppressive antibiotics. Trends in CRP were compared between two groups: failed and successful DAIR. Receiver operating characteristic (ROC) curves were analysed.

**Results:**

189 DAIR procedures were included, of which 49% (92) failed and 51% (97) were successful. Mean follow-up was 7.5 years. Overall, CRP trended down following surgery. Mean CRP was significantly higher in the failed DAIR group at all time points. The greatest difference was at week one post-operatively (mean 76 vs. 101, *P* < 0.01). This remained significant when patients experiencing DAIR failure within 90 days were excluded (mean 76 vs. 107, *P* < 0.01). CRP was most accurate as a predictor of failure at week one post-operatively, with an area under the curve (AUC) of 0.71. Optimal balance of specificity and sensitivity was achieved with a CRP cutoff of 89. This yielded a sensitivity of 74% and specificity of 66%. CRP at week one post-operatively more accurately predicted DAIR failure than admission CRP.

**Conclusions:**

CRP measured at week one post-operatively demonstrated moderate prognostic value for predicting DAIR failure in the treatment of total knee PJI. While an elevated CRP may assist in identifying patients who require close monitoring, CRP alone is insufficient to guide decisions around revision surgery.

## Introduction

Periprosthetic joint infection (PJI) remains the leading cause of reoperation following total knee arthroplasty (TKA) [1–5]. It is a devastating complication associated with significant morbidity, loss of income, psychological stress, hospitalisation and health expenditure [6]. Open debridement, antibiotics and implant retention (DAIR) surgery is frequently performed as a first-line treatment for PJI [7–8]. Variable success rates have been reported, ranging from 20 to 85% [1, 8–15]. Timely identification of patients at risk of DAIR failure is essential to guide appropriate treatment planning and prevent avoidable morbidity. While different scoring systems have been developed to predict risk of DAIR failure, external validation has revealed that these have variable efficacy and cannot be relied upon in isolation [16–20].

C-reactive protein (CRP) is an acute phase reactant produced by the liver in response to systemic infection or inflammation [21–22]. Serum CRP is a minor criterion in the revised International Consensus Meeting definition of PJI [23]. Its efficacy as a diagnostic test in PJI has been thoroughly investigated, with sensitivity reported to range between 62 and 100%, and specificity between 64 and 94% [21, 24–28]. It is readily accessible, low cost and less invasive than other diagnostic tools such as synovial alpha defensin or tissue biopsy [24, 29]. As a result, it is widely used as a serum biomarker in the diagnosis and monitoring of PJI.

While the use of CRP as a tool to help determine the optimal timing for reimplantation in two stage revision surgery has been investigated, there is scant evidence regarding its ability to predict DAIR failure post-operatively [30–34]. The aim of this study was to determine the accuracy of post-operative serum CRP as a predictor of DAIR failure in total knee PJI. By analysing trends in post-operative CRP, we hope to determine whether it is a reliable tool to augment decision making in the early post-operative course.

## Methods

This is a multicentre retrospective cohort study encompassing all patients treated at three tertiary level hospitals in Auckland, New Zealand, between 1 January 2000 and 31 December 2015. All patients who underwent DAIR surgery for first episode total knee PJI were included. The definition of PJI was based on the Musculoskeletal Infection Society consensus statement [1]. Patients younger than 18 years old, and those with negative tissue cultures following surgery were excluded. All three institutions followed similar treatment protocols. DAIR was performed through the same approach as the primary surgery. Exchange of liner was carried out where possible, but not in patients with an all-polyethylene tibia, or where components were not readily available. Post-operative antibiotic regimes were determined in conjunction with an infectious disease specialist.

Audits of clinical coding from operating theatres and discharge summaries were used to identify patients. These were cross referenced to records from The New Zealand Joint Registry. If the diagnosis was not clear, physical notes were reviewed and a consensus reached between authors. Baseline characteristics were collected to define patient demographics including age, sex, body mass index (BMI), American Society of Anaesthesiologists (ASA) score, smoking status, ethnicity and comorbidities. Dates of primary TKA, DAIR and any subsequent surgeries were obtained for each patient.

Electronic laboratory records (Sysmex Éclair) were accessed to collect CRP levels upon presentation, immediately prior to DAIR surgery, and at 1, 2, 4 and 6 weeks post-operatively. Tissue culture results were also collected. Patients were then divided into two groups: successful DAIR (S-DAIR) and failed DAIR (F-DAIR). The definition of failed DAIR was based on the International Consensus Meeting (ICM) definition of infection control following staged revision, and included at least one of the following criteria: (1) death related to PJI, (2) reinfection confirmed with at least 1 positive aspirate or intra-operative sample, (3) revision surgery for any cause excluding trauma, with repeat DAIR performed more than 7 days following initial DAIR considered as failure, and (4) requirement for long-term suppressive antibiotics at follow-up to control infection, as decided by an orthopaedic surgeon and infectious disease physician [35]. Long-term suppressive antibiotic therapy was defined as a documented plan for lifelong antibiotics from an infectious disease or orthopaedic specialist at latest follow-up.

The two groups were then compared. Continuous variables are presented as mean ± standard deviation and categorical variables as percentages. Univariate analysis of continuous data was performed using independent t-test for normally distributed data, and Mann-Whitney U test for non-normally distributed data. Chi square or Fisher’s exact tests were used for categorical data. Multivariate logistic regression was performed to identify factors associated with DAIR failure. Variables with a P-value < 0.20 on univariate analysis or strong clinical relevance were included. Multicollinearity between candidate predictors was assessed using the variance inflation factor (VIF), with a threshold of 3 indicating significant collinearity. Missing data was handled with pairwise deletion. In all comparisons a P-value < 0.05 was considered statistically significant. Post-operative CRP levels were compared at different time points. Receiver operating characteristic (ROC) curves were analysed to determine the efficacy of CRP as a tool for predicting DAIR failure.

## Results

### Demographics

189 patients were included, of which there were 97 (51%) S-DAIR and 92 (49%) F-DAIR. Age, sex, ethnicity, comorbidities, smoking status, BMI, ASA score and symptom duration were similar between groups. (Table 1) Mean time from primary joint replacement to DAIR surgery was significantly lower in the S-DAIR group (1.3 vs. 2.9 years, *P* < 0.01). Mean follow-up was 7.5 years. Most patients presented shortly after symptom onset, with 84% (158) presenting within 14 days.

74% of patients in the F-DAIR group subsequently underwent a first stage revision, and 60% went on to second stage revision. 20% were treated with long-term suppressive antibiotics. Among F-DAIR patients who proceeded to revision surgery, the mean time between DAIR and revision surgery was 98 days. The average time to DAIR failure was 114 days.

The most common primary organism was MSSA (methicillin susceptible Staphylococcus aureus), isolated in 34% of patients. 73% of CoNS (coagulase negative Staphylococcus) cases were in the S-DAIR group (*P* = 0.03). Of the 37 patients with polymicrobial infections, 68% were in the S-DAIR group (*P* = 0.03).

**Table 1:** Patient demographics grouped by DAIR success

	Success	Failure	*P*-value
Total (*N*)	97	92	
Age (mean ± SD)	67.2 ± 10.6	68.8 ± 11.4	0.32
Sex (female)	38% (37)	35% (32)	0.63
Ethnicity			
NZ European	71% (69)	73% (67)	0.69
Pacific	16% (16)	13% (12)	
Māori	7% (7)	11% (10)	
Asian	5% (5)	3% (3)	
Comorbidities			
DM	22% (21)	22% (20)	0.99
RA	7% (7)	8% (7)	0.92
IS	6% (6)	5% (5)	0.83
CKD	9% (8)	8% (8)	0.91
Current smoker	9% (7)	12% (12)	0.48
BMI (mean ± SD)	34.5 ± 8.7	33.6 ± 8.7	0.52
ASA (I-II)	47% (46)	57% (52)	0.21
Symptom duration, days (mean ± SD)	10.9 ± 17.6	13.9 ± 28.8	0.41
Prosthetic age, years (mean ± SD)	1.3 ± 2.4	2.9 ± 3.8	**< 0.01**
Follow-up, years (mean ± SD)	7.6 ± 3.8	7.6 ± 3.9	0.96
Polymicrobial infection	68% (25)	32% (12)	**0.03**
Primary organism			
MSSA	45% (29)	55% (36)	0.18
MRSA	50% (3)	50% (3)	0.99
CoNS	79% (27)	21% (7)	**< 0.01**
*Streptococcus*	46% (22)	54% (26)	0.38
*E. coli*	56% (5)	44% (4)	0.99
Other Gram positive	44% (4)	56% (5)	0.99
Other Gram negative	39% (7)	61% (11)	0.27

Statistically significant results in bold

DM: diabetes mellitus. RA: rheumatoid arthritis. IS: immunosuppressed. CKD: chronic kidney disease. BMI: body mass index. ASA: American Society of Anaesthesiologists score. Prosthetic age: time from primary TKA to DAIR. MSSA: methicillin susceptible *Staphylococcus aureus*. MRSA: methicillin resistant *Staphylococcus aureus*. CoNS: coagulase negative Staphylococcus

### Inflammatory markers and DAIR success

**Fig. 1:**
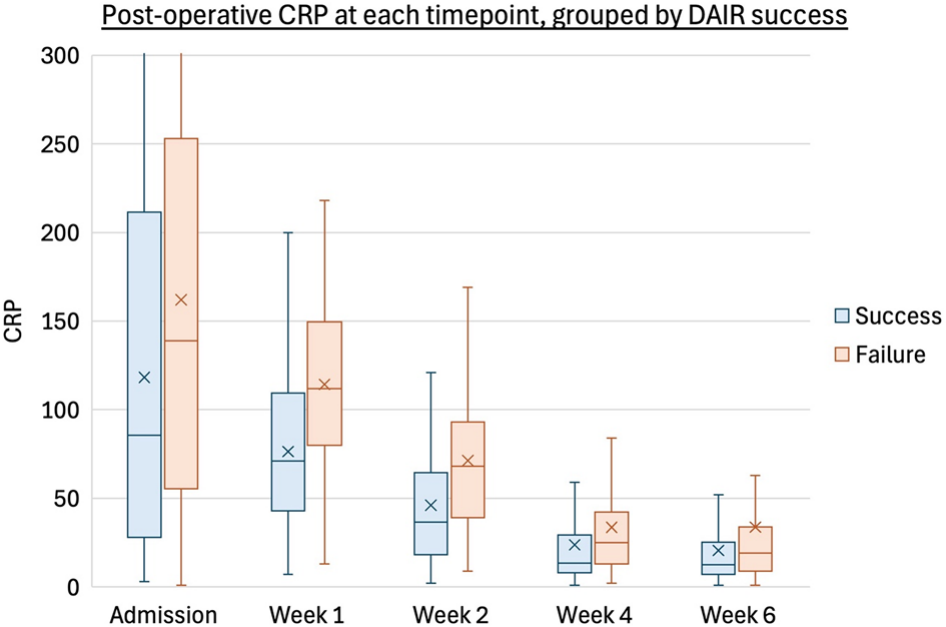
Post-operative CRP at each timepoint, grouped by DAIR success

CRP at admission was significantly lower for patients in the S-DAIR group (mean 118 vs. 162, *P* = 0.03) (Table 2). There were no significant differences in WBC, ESR or ESR: CRP ratio (ECR) at admission. Overall, CRP trended down for both groups in the post-operative period (Fig. 1). Mean CRP was significantly lower in the S-DAIR group at all post-operative time points. Difference in mean CRP between groups decreased as time from DAIR surgery increased. The greatest difference was at week one (76 vs. 101 mg/L, *P* < 0.01). At week six, the mean CRP in S-DAIR was 21, compared to 34 mg/L in F-DAIR (*P* < 0.01).

The difference in week one CRP remained significant when patients who experienced DAIR failure within 42 days (mean 76 vs. 101 mg/L, *P* < 0.01) and 90 days (mean 76 vs. 107 mg/L, *P* < 0.01) were excluded. Subgroup analysis revealed week one CRP was significantly lower in S-DAIR for acute post-operative (66 vs. 112 mg/L, *P* < 0.01) and acute haematogenous (84 vs. 117 mg/L, *P* < 0.01) infections. There was no significant difference between groups among chronic infections (79 vs. 101 mg/L, *P* = 0.33). Given the use of long-term suppressive antibiotics is subjective, a sensitivity analysis was performed including these patients in the S-DAIR group. The difference in week one CRP increased (mean 81 vs. 117, *P* < 0.01).

**Table 2:** Inflammatory markers by DAIR success

	Success	Failure	*P*-value
Admission (mean ± SD)			
CRP (mg/L)	118 ± 102	162 ± 126	**0.03**
ESR (mm/h)	64 ± 35	63 ± 36	0.94
ECR	1.7 ± 2.6	1.1 ± 2.2	0.24
WBC (x10^9/L)	12.1 ± 7.4	16.0 ± 30.2	0.21
Post-operative CRP (mean ± SD)			
Week 1	76 ± 44	114 ± 55	**< 0.01**
Week 2	46 ± 36	71 ± 43	**< 0.01**
Week 4	24 ± 25	34 ± 30	**< 0.01**
Week 6	21 ± 24	34 ± 41	**0.04**
Week 1 CRP by group (mean ± SD)			
Acute post-operative	66 ± 41 (44)	112 ± 66 (23)	**< 0.01**
Acute haematogenous	84 ± 46 (48)	117 ± 54 (60)	**< 0.01**
Chronic	79 ± 40 (5)	101 ± 35 (9)	0.33
Week 1 CRP by time to failure (mean ± SD)			
Before 42 days	76 ± 44	130 ± 58 (46)	**< 0.01**
After 42 days	76 ± 44	101 ± 50 (46)	**< 0.01**
Before 90 days	76 ± 44	118 ± 62 (63)	**< 0.01**
After 90 days	76 ± 44	107 ± 40 (29)	**< 0.01**

Statistically significant results in bold

ECR: ESR to CRP ratio. WBC: white blood cell count. CRP values in mg/L. Where relevant, numbers in parentheses indicate the number of patients in each subgroup

### Diagnostic value and optimal threshold

**Fig. 2:**
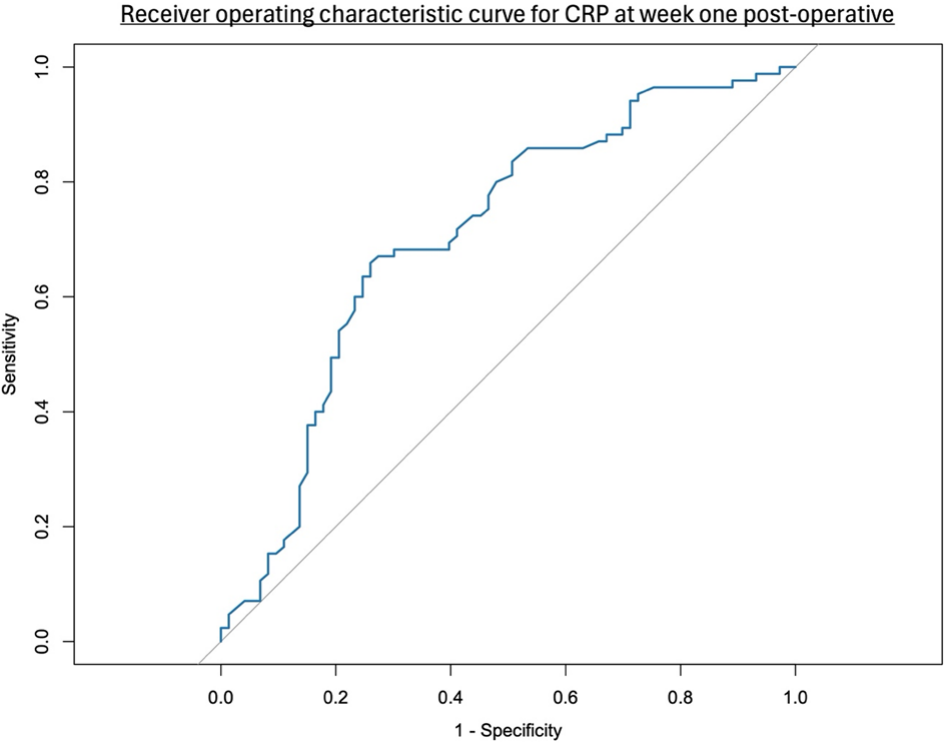
ROC curve for CRP at week one post-operative

ROC curve analysis of week one CRP yielded an area under the curve (AUC) of 0.71 (95% CI 0.61–0.81). The CRP threshold with the optimal balance of sensitivity and specificity was 89 mg/L. This yielded a sensitivity of 74% (95% CI 64–84%) and a specificity of 66% (95% CI 56–75%). In comparison, CRP at admission had an AUC of 0.59, with an optimal cut point of 102 mg/L. This yielded a sensitivity of 64% and specificity of 56% (Fig. 2) (Table 3).

**Table 3:** Multivariate analysis of factors predicting DAIR success

	Odds ratio (success)	Confidence interval	*P*-value
Week one CRP < 89 mg/L	5.7	2.3–14.6	< 0.01
Prosthetic age > 28 days	0.2	0.1–0.9	**0.03**
Any comorbidity	0.76	0.3–2.0	0.58
Current smoker	0.6	0.1–3.5	0.59
BMI > 30	1.0	0.4–2.7	0.99
Polymicrobial infection	0.7	0.2–2.4	0.53
Primary organism (vs. MSSA)			
MRSA	1.9	0.1–46.4	0.70
CoNS	2.0	0.4–9.4	0.36
*Streptococcus*	1.2	0.4–3.7	0.81
*E. coli*	2.7	0.5–15.9	0.28
Other Gram positive	0.4	0.1–4.3	0.44
Other Gram negative	0.2	0.1–1.5	0.13

Statistically significant results in bold

Any comorbidity includes diabetes mellitus, rheumatoid arthritis, immunosuppression and chronic kidney disease

### Multivariate analysis

Multivariate analysis determined the odds of DAIR surgery being successful were 5.7 times greater in patients with a week one post-operative CRP value less than 89 mg/L (95% CI 2.3–14.6, *P* < 0.01). Prosthetic age greater than 28 days was associated with decreased odds of DAIR success (OR 0.2, 95% CI 0.1–0.9). No other variables were significantly associated with DAIR success in this cohort. Presence of any comorbidity, smoking status and BMI greater than 30 were included due to their established role as risk factors for PJI. No evidence of multicollinearity was observed.

## Discussion

This study demonstrates that early post-operative CRP is a better predictor of DAIR failure than admission CRP, in patients undergoing DAIR for total knee PJI. Week one post-operative CRP was strongly associated with DAIR success. Patients with a week one post-operative CRP greater than 89 mg/L were five times as likely to experience DAIR failure. Overall, 51% of DAIR procedures were successful. As in previous studies, we found that age of prosthesis was a significant independent risk factor for DAIR failure.

Although the association between week one CRP and DAIR failure was statistically significant, the diagnostic performance of this marker must be interpreted cautiously. A CRP threshold of 89 mg/L at week one demonstrated a sensitivity of 74% and specificity of 66%. This indicates that a substantial proportion of false positives and false negatives would occur if CRP were used as a standalone diagnostic test in clinical practice. The positive predictive value was 67% and the negative predictive value 74%, suggesting only moderate discriminative ability. The AUC of 0.71 further indicates moderate prognostic performance. While this represents an improvement compared with admission CRP, it is insufficient to guide surgical decision-making in isolation. CRP is a non-specific inflammatory marker that may remain elevated following surgery due to tissue trauma, systemic inflammatory responses, or other concurrent conditions [21, 22]. Consequently, week one CRP should be interpreted as a complementary marker within a broader clinical context, rather than as an isolated trigger for revision surgery.

Beyond week one, the predictive value of CRP diminished. Although mean CRP values remained significantly higher in the F-DAIR group at all time points, the difference between groups was relatively small. This suggests that the routine use of serial CRP measurements during antibiotic therapy provides limited additional diagnostic value. CRP may instead be more useful in monitoring recurrence of infection after completion of antibiotic therapy, when inflammatory markers would otherwise be expected to normalise.

We found no association between week one CRP and treatment success in cases of chronic PJI. However, only a small number of chronic infections were included in this cohort. Given that staged revision with component exchange is typically performed as a first-line treatment for chronic PJI, this finding is not unexpected. In the chronic cases included in this study, patient or clinical factors often precluded staged revision surgery, and DAIR was performed as an alternative treatment strategy.

There is limited published data evaluating the role of post-operative CRP in predicting DAIR failure. In a cohort of 51 hips and 50 knees undergoing DAIR, Bejon et al. reported that CRP did not predict treatment failure and recommended against routine CRP monitoring during treatment of PJI. However, their definition of failure did not include death or long-term suppressive antibiotic therapy, which may have resulted in underestimation of failure rates [36]. Other studies have evaluated CRP as a prognostic marker in staged revision surgery. Benda et al. reported that a CRP threshold of 11.35 mg/L following first stage revision arthroplasty demonstrated a sensitivity of 75% and specificity of 54% in predicting success of the second stage [37]. Similarly, a meta-analysis investigating CRP as a predictor of success after staged revision surgery reported an overall sensitivity of 45% and specificity of 73% [38]. These findings highlight the considerable variability in reported CRP thresholds and diagnostic performance across different treatment strategies for PJI.

Maier et al. evaluated the erythrocyte sedimentation rate (ESR) to CRP ratio (ECR) as a predictor of reinfection following DAIR surgery. In their cohort of 179 patients, admission ECR predicted acute postoperative reinfection with an AUC of 0.62, sensitivity of 83%, and specificity of 49%. Admission CRP alone demonstrated an AUC of 0.64 [39]. In comparison, our findings suggest that week one post-operative CRP (AUC 0.71) is a more accurate predictor of treatment success than admission CRP or ECR. Diagnostic performance in our study was best for acute haematogenous PJI.

Scoring systems have been developed to estimate the risk of DAIR failure. The KLIC and CRIME80 scores have been proposed for acute post-operative and acute haematogenous PJI, respectively [16–20]. However, external validation studies have demonstrated relatively poor predictive performance, with reported AUC values of approximately 0.51 for KLIC (failure at 90 days) and 0.61 for CRIME80 [17, 19–20, 40]. Both the KLIC and CRIME80 scores include pre-operative CRP. Although the diagnostic performance of week one CRP in our study appears superior to these scoring systems, an AUC of 0.71 still indicates only moderate predictive accuracy. Further research could look to assess whether week one post-operative CRP could improve the diagnostic accuracy of these tools.

DAIR failure is associated with increased morbidity, highlighting the importance of early identification of patients at risk of treatment failure [40]. The findings of this study suggest that early post-operative CRP may provide useful adjunctive information when assessing patients following DAIR surgery. In clinical practice, a persistently elevated CRP at one week post-operatively may prompt closer clinical surveillance, earlier multidisciplinary discussion, or consideration of further investigation for persistent infection. Given the moderate diagnostic accuracy observed in this study, CRP should not be used in isolation to guide decisions regarding revision surgery. Rather, CRP values should be interpreted alongside clinical progress and host factors.

We acknowledge there are limitations to this study. First, this study was retrospective and treatment decisions were made by individual surgeons across multiple institutions. Although clinical practice was broadly similar between hospitals, variability in surgical decision-making, antibiotic protocols, and post-operative management may have influenced both CRP trajectories and treatment outcomes. To limit variability, included only patients with confirmed PJI following primary total knee arthroplasty and positive tissue cultures, which improves diagnostic certainty. The use of a centralised electronic patient record across the three participating hospitals enabled reliable identification and follow-up of patients. In addition, linkage with the New Zealand Joint Registry allowed identification of staged revision procedures performed outside of the participating institutions. Second, the timing and frequency of CRP measurements may have varied between institutions and individual clinicians, potentially introducing measurement variability. While most patients underwent routine post-operative CRP testing, adherence to standardised testing intervals could not be guaranteed. Third, although multivariate logistic regression was performed, the number of variables included relative to the number of outcome events introduces a potential risk of model overfitting. Furthermore, although important confounders such as infection chronicity and microbiological profile were considered, residual confounding may still be present. Finally, the exclusion of culture-negative infections may limit the generalisability of these findings to the broader population of patients undergoing treatment for PJI. Culture-negative PJI represents a clinically relevant subgroup in which inflammatory markers might behave differently.

## Conclusion

CRP measured at week one post-operatively demonstrated moderate prognostic value for predicting DAIR failure in the treatment of total knee PJI. While an elevated CRP may assist in identifying patients who require close monitoring, CRP alone is insufficient to guide decisions around revision surgery.

## Data Availability

No datasets were generated or analysed during the current study.
